# Outcomes of Nephrolithiasis Care by Patients' Race: From a Statewide Quality Improvement Collaborative

**DOI:** 10.7759/cureus.108310

**Published:** 2026-05-05

**Authors:** Brianna A Guillen, Fumihiko Nakamura, Brandon Dodd, Ademilola Tejuoso, Sarah Johnson, Eric Wahlstedt, Andrew Harris, Emily Slade, Caitline Phan, Kellen Choi

**Affiliations:** 1 Medicine, University of Texas Rio Grande Valley School of Medicine, Edinburg, USA; 2 Medicine, University of Louisville School of Medicine, Louisville, USA; 3 Department of Urology, Brookwood Baptist Health, Homewood, USA; 4 Department of Urology, University of Louisville Hospital, Louisville, USA; 5 Department of Emergency Medicine, Cook County Health, Chicago, USA; 6 Medicine, University of Kentucky College of Medicine, Lexington, USA; 7 Department of Urology, University of Kentucky, Lexington, USA; 8 Department of Biostatistics, University of Kentucky, Lexington, USA

**Keywords:** nephrolithiasis care, population health management, quality improvement, racial disparities, social determinants of health

## Abstract

Objectives

Kidney stone disease, or nephrolithiasis, is a condition that is prevalent amongst the population in the United States. The state of Kentucky is part of the “Kidney Stone Belt”, which has the highest prevalence of kidney stones in the United States. Although kidney stone formation is multifactorial, efforts must be made by urologists in the Kidney Stone Belt to provide consistent care that is in accordance with American Urological Association (AUA) guidelines. To implement quality improvement measures in nephrolithiasis care, our study first aims to reveal inadequacies of our approach to care today.

Methods

We completed retrospective chart reviews of patients with nephrolithiasis from 13 urologists affiliated with two academic medical institutions. Individual data points were collected for each patient and stored in a database. The data was analyzed and tested for statistical significance using the Chi-squared test and stratified by race.

Results

Upon analysis of our data, we noted statistically significant racial differences in the following areas of nephrolithiasis care: post-lithotripsy stone analysis, orders for 24-hour urine tests, and dietary histories. Although not statistically significant, our population, regardless of race, had little to no documentation of physician-led counseling for kidney stone prevention.

Conclusions

Our analysis identifies several areas where patients could benefit from quality improvement measures in nephrolithiasis care. We recommend adherence to AUA guidelines for the medical management of nephrolithiasis care to prevent compromise of the quality of care provided to racial minority patients. Additionally, social determinants of health may have a role to play in disparities noted in this study.

## Introduction

Kidney stone disease, or nephrolithiasis, is one of the most common conditions that affects the kidneys of people in the United States [1]. Nephrolithiasis results in high health care costs in the United States; specifically, in the United States, nephrolithiasis care costs more than 2 billion dollars annually [2]. In the United States, one in 11 people have a history of nephrolithiasis [3]. However, both incidence and prevalence of kidney stones continue to increase across the world [4]. Nephrolithiasis is a complex and multifactorial disease. Studies have found correlations between the development of nephrolithiasis in males, increased triglycerides, diabetes, gallstone disease, white race, and ZIP code [5]. However, lower income, nonwhite race, unemployment, female gender, and a BMI over 40 kg/m^2 ^are all factors that have been associated with a lower quality of life (QoL) in patients with nephrolithiasis [6].

Historically, minority patients have typically experienced lower quality of health care. This was officially documented in the Institute of Medicine’s 2003 report “Unequal Treatment: Confronting Racial and Ethnic Disparities in Health Care”. This report determined that access to care may result from varying socioeconomic circumstances. However, when these circumstances are accounted for, race and ethnicity are highly likely to play a significant role in the quality of health care that is received by a patient [7]. Furthermore, several studies have identified disparities in various aspects of racial and ethnic minority patient care, particularly in Black and Hispanic patients. A comprehensive review of National Hospital Ambulatory Medical Care Survey (NHAMCS) data found that white patients were more likely to be prescribed an opioid than Black and Hispanic patients during pain-related visits [8]. Disparities in nephrolithiasis care have also been noted in numerous studies. One statewide study discovered that Black and Hispanic patients had an average longer time to surgery than White patients in the state of California. Black patients experienced 36% longer wait times, while Hispanic patients experienced 20% longer wait times [9].

The role that race plays in the risk of kidney stone formation is not well understood. Few studies have analyzed the correlation between race and kidney stone disease; however, recent studies have shown that white patients are at a higher risk for developing nephrolithiasis than Black patients [10]. The “Kidney Stone Belt” of the United States is a region, spanning from Kansas to North Carolina, where the prevalence of nephrolithiasis is highest. High temperatures in this region increase the likelihood of fluid loss, leading to increased urinary saturation; additional regional factors, including dietary patterns such as sugar-sweetened beverage consumption, may further influence stone risk. Increased urinary saturation directly increases the likelihood of stone formation [11].

As a member of this region, citizens of Kentucky are at higher risk of developing nephrolithiasis. Therefore, nephrolithiasis patients in the state of Kentucky would greatly benefit from the analysis of the quality of nephrolithiasis care provided by urologists in the state. Although there are various factors that can influence the likelihood of stone formation, such as socioeconomic status, climate, and race, efforts should be made to provide and ensure nephrolithiasis care that is consistent and in line with AUA guideline standards for the medical management of kidney stones. Given the retrospective and exploratory nature of this analysis, findings were intended to identify patterns of care rather than establish causal relationships. This quality improvement-focused, retrospective observational study aims to evaluate adherence to the American Urological Association guideline-recommended components of nephrolithiasis care, including dietary history documentation, 24-hour urine metabolic testing, and stone analysis, and to assess whether racial differences exist in the delivery of these elements among patients treated at two academic medical institutions in Kentucky with non-overlapping catchment areas. With this knowledge, we can further discuss potential options for improving care for all patients.

## Materials and methods

Patient population 

We conducted a retrospective chart review of 429 nephrolithiasis patients who received care from 13 different urologists between January 2017 and February 2022. Inclusion criteria for our patients included adults, aged 18 and over, who were diagnosed with nephrolithiasis between January 2017 and February 2022 at two academic medical institution-affiliated clinical sites in Kentucky with non-overlapping catchment areas. All patients in our study were evaluated by 13 urologists in Kentucky. Exclusion criteria included pediatric patients aged 18 or younger. The initial data extract contained 485 patients, and 44 patients were excluded because they were under the age of 18. The remaining 441 adult patients met the criteria for the study, and 12 of these patients (2.7%) were excluded due to having incomplete data in the demographic variables of age, gender, and race. The finalized analytic dataset included 429 patients. Available case analysis was used for all analyses.

Data 

During patient visits, 105 data points were collected for each patient. The Research Electronic Data Capture (REDCap) system was used to form a database between the two academic medical institutions. A CPT code of 52353 was used to search for nephrolithiasis cases between January 2017 and February 2022. The clinical variables that were collected included: patient discharge with opioids (Yes/No), emergency department visit (Yes/No), patient hospitalization (Yes/No), documentation of dietary history (Yes/No), parathyroid hormone testing (Yes/No), post-lithotripsy stone removal (Yes/No), stone composition analysis (calcium oxalate monohydrate/calcium oxalate dihydrate/calcium phosphate/uric acid/struvite/cystine/other), orders for 24 hour urine test (Yes/No), 24 hour urine test obtained (Yes/No), patient counseling on 2.5 L of urine output (Yes/No), patient counseling on low non-dairy animal protein diet (Yes/No), patient counseling on low sodium diet (Yes/No), and patient counseling on low oxalate diet (Yes/No). Demographic variables included age, gender (female, male, other), and race. Race was recorded as specified in the electronic health records, including the following categories: American Indian or Alaska Native, Asian, Black or African American, White, and Other patients.

Outcomes and data analysis

Clinical characteristics were summarized overall and stratified by self-reported racial group. To assess differences in nephrolithiasis care by racial group, clinical characteristics were compared across racial groups using Fisher’s exact tests. To address small cell counts in these analyses, racial groups were categorized as Black or African American, White, and Other patients, where Other includes Asian, American Indian or Alaskan Native patients, and any race categorized as “Other” in the electronic health record. Statistically significant differences were defined by differences that yielded a p-value of less than 0.05. No adjustment was made for multiple testing, as this was an exploratory analysis. Analyses were performed using R version 4.2.0.

## Results

Our patient population consisted of a total of 429 patients. The mean age of our patient population was 50.0 years (standard deviation, SD=14.9). Of our patient population, 53.1% were female, and 46.9% were male. The racial breakdown for this patient population included 359 White individuals (83.7%), 55 Black or African American individuals (12.8%), and 15 individuals in other racial minority groups (3.5%), including Asian, American Indian or Alaska Native, and any race categorized as “Other” in the electronic health record (Table 1). Upon stratification of our data by race, we found racial disparities in the following aspects of nephrolithiasis care: post-lithotripsy stone analysis, orders for 24-hour urine tests, and dietary histories. The proportion of patients having a dietary history taken during their initial evaluation was 12.3 percentage points lower among Black or African American patients (5.5%) compared to White patients (17.8%).

**Table 1:** Stratification of patient data by racial groups Missing data in each variable is indicated by “Unknown.” Variables without “unknown” listed have no missing data. Racial minority patient groups in the category Other include: Asian, American Indian or Alaskan Native, and any other race listed as “Other” in the electronic health record. P-values were calculated using Fisher’s exact tests. * indicates p<0.05.

Characteristics	Overall (N = 429)	Black or African American Patients (N = 55)	White Patients (N = 359)	Other Patients (N = 15)	p-value
Visited Emergency Department					0.108
No	346 (80.7%)	43 (78.2%)	294 (81.9%)	9 (60.0%)	
Yes	83 (19.3%)	12 (21.8%)	65 (18.1%)	6 (40.0%)	
Patient Hospitalized					0.520
No	374 (87.2%)	47 (85.5%)	315 (87.7%)	12 (80.0%)	
Yes	55 (12.8%)	8 (14.5%)	44 (12.3%)	3 (20.0%)	
Patient Discharged on Opioids					0.058
No	341 (79.5%)	37 (67.3%)	291 (81.1%)	13 (86.7%)	
Yes	88 (20.5%)	18 (32.7%)	68 (18.9%)	2 (13.3%)	
Dietary History Taken					0.022*
No	358 (83.4%)	52 (94.5%)	295 (82.2%)	11 (73.3%)	
Yes	71 (16.6%)	3 (5.5%)	64 (17.8%)	4 (26.7%)	
Parathyroid Hormone Tested					0.091
No	367 (85.5%)	52 (94.5%)	302 (84.1%)	13 (86.7%)	
Yes	62 (14.5%)	3 (5.5%)	57 (15.9%)	2 (13.3%)	
Stone Analysis Performed					0.019*
No	112 (32.9%)	23 (51.1%)	86 (30.6%)	3 (21.4%)	
Yes	228 (67.1%)	22 (48.9%)	195 (69.4%)	11 (78.6%)	
Unknown	89	10	78	1	
24-hour Urine Test Ordered					<0.001*
No	305 (71.1%)	52 (94.5%)	241 (67.1%)	12 (80.0%)	
Yes	124 (28.9%)	3 (5.5%)	118 (32.9%)	3 (20.0%)	
24-hour Urine Test Obtained					<0.001*
No	50 (30.7%)	15 (88.2%)	33 (23.2%)	2 (50.0%)	
Yes	113 (69.3%)	2 (11.8%)	109 (76.8%)	2 (50.0%)	
Unknown	266	38	217	11	
Counseled on Achieving 2.5L Daily Urine Output					0.742
No	217 (86.8%)	21 (87.5%)	187 (86.2%)	9 (100.0%)	
Yes	33 (13.2%)	3 (12.5%)	30 (13.8%)	0 (0.0%)	
Unknown	179	31	142	6	
Counseled on Low Non-Dairy Animal Protein Diet					0.795
No	134 (91.2%)	12 (100.0%)	115 (89.8%)	7 (100.0%)	
Yes	13 (8.8%)	0 (0.0%)	13 (10.2%)	0 (0.0%)	
Unknown	282	43	231	8	
Counseled on Low Sodium Diet					0.223
No	126 (86.3%)	12 (100.0%)	107 (84.3%)	7 (100.0%)	
Yes	20 (13.7%)	0 (0.0%)	20 (15.7%)	0 (0.0%)	
Unknown	283	43	232	8	
Counseled on Low Oxalate Diet					0.560
No	128 (89.5%)	12 (100.0%)	109 (87.9%)	7 (100.0%)	
Yes	15 (10.5%)	0 (0.0%)	15 (12.1%)	0 (0.0%)	
Unknown	286	43	235	8	

Furthermore, the rate of post-lithotripsy stone analysis was also 20.5 percentage points lower among Black or African American patients (48.9%) than among White patients (69.4%) (Table 2). Lastly, the proportion of patients having a 24-hour urine analysis ordered was 27.4 percentage points lower among Black or African American patients (5.5%) compared to White patients (32.9%). Each of these differences, shown in Table 1, was statistically significant (p<0.05). Although there were no statistically significant differences by race, our patient population, across all races, had little to no documentation of physician-led counseling on measures to reduce kidney stone recurrence.

**Table 2:** Stone analysis results for patients with tested kidney stone Patients with a kidney stone analysis included (n = 228). There was no missing data in any of the presented variables. Racial minority patient groups in the category Other include: Asian, American Indian or Alaskan Native, and any other race listed as “Other” in the electronic health record. Stone analysis characteristics denoted as “Other” include kidney stones of a combination of various compositions. P-values were calculated using Fisher’s exact tests. * indicates p<0.05.

Characteristic	Overall (N = 228)	Black or African American Patients (N = 22)	White Patients (N = 195)	Other Patients (N = 11)	p-value
Calcium Oxalate Monohydrate					0.404
No	73 (32.0%)	5 (22.7%)	66 (33.8%)	2 (18.2%)	
Yes	155 (68.0%)	17 (77.3%)	129 (66.2%)	9 (81.8%)	
Calcium Oxalate Dihydrate					0.122
No	129 (56.6%)	16 (72.7%)	109 (55.9%)	4 (36.4%)	
Yes	99 (43.4%)	6 (27.3%)	86 (44.1%)	7 (63.6%)	
Calcium Phosphate					0.015*
No	136 (59.6%)	19 (86.4%)	112 (57.4%)	5 (45.5%)	
Yes	92 (40.4%)	3 (13.6%)	83 (42.6%)	6 (54.5%)	
Uric Acid					0.277
No	210 (92.1%)	22 (100.0%)	177 (90.8%)	11 (100.0%)	
Yes	18 (7.9%)	0 (0.0%)	18 (9.2%)	0 (0.0%)	
Ammonium Magnesium Phosphate (Struvite)					0.467
No	224 (98.2%)	21 (95.5%)	192 (98.5%)	11 (100.0%)	
Yes	4 (1.8%)	1 (4.5%)	3 (1.5%)	0 (0.0%)	
Cystine					>0.999
No	227 (99.6%)	22 (100.0%)	194 (99.5%)	11 (100.0%)	
Yes	1 (0.4%)	0 (0.0%)	1 (0.5%)	0 (0.0%)	
Other					>0.999
No	220 (96.5%)	22 (100.0%)	187 (95.9%)	11 (100.0%)	
Yes	8 (3.5%)	0 (0.0%)	8 (4.1%)	0 (0.0%)	

## Discussion

Overall, dietary histories, 24-hour urine test orders and results, and stone analyses were less often taken for Black or African American patients. Without these crucial aspects of the standard nephrolithiasis workup, health care providers have less information to work with to construct a care plan following stone removal. Therefore, it is important for health care providers to review the standard guidelines for the continuum of nephrolithiasis care, as outlined by the American Urological Association.

AUA guidelines for the medical management of kidney stones

The AUA provides a guideline framework for the continuum of care of adult patients with kidney stones. In the evaluation stage of care, the AUA recommends taking detailed medical and dietary histories, serum chemistries, and urinalysis for all patients. Additionally, stone analyses should be obtained if the stone is available. Relevant images available for patients should be assessed for quantification of stone burden [12]. 

A critical component of nephrolithiasis care, as described in the AUA guidelines, is diet therapy, which includes counseling by a health care provider. Diet therapy includes counseling a patient about the proper diet to follow, depending on the composition of the stone. For instance, patients with calcium oxalate stones and high urinary oxalate should be counseled to reduce their oxalate intake and maintain a normal calcium intake. For patients with stones composed of uric acid or patients with calcium stones and a relatively high level of urinary uric acid, patients should be counseled to limit their consumption of non-dairy animal protein. Patients with cystine stones should be counseled to limit their sodium and protein intake. Patients with calcium stones and high levels of urinary calcium should be counseled to reduce their sodium intake and have a dietary calcium intake of 1000 - 1200 mg per day. Lastly, patients with calcium stones and low urinary citrate should be counseled to increase their intake of vegetables and fruits and limit their intake of non-dairy animal protein [12]. For all stone formers, patients should be counseled to have a fluid intake that will produce a daily urine volume of 2.5 liters [12]. With counseling, patients are better able to play an active role in their care plan and make lifestyle and dietary changes to decrease the likelihood of stone recurrence. 

Dietary history, 24-hour urine tests, and stone analysis

Taking a detailed dietary history is a key part of kidney stone care. Although genetics may influence an individual’s susceptibility to developing kidney stones, environmental factors also play a major role in the development of kidney stones [13]; therefore, dietary histories are useful in providing information about a stone former’s nutritional risk factors. With information on these nutritional risk factors, health care providers can more effectively counsel patients on which foods to prevent stone recurrence. In our patient population, African American patients did not have their dietary histories taken as consistently as our White patients. This increases African American patients’ susceptibility to experiencing stone recurrence.

Twenty-four-hour urine tests, which are typically collected at home, are also part of the standard for nephrolithiasis care [12]. Twenty-four-hour urine tests are metabolic evaluations that help health care providers in providing the proper dietary and pharmacologic therapies to prevent stone recurrence [14]. Our results showed lower rates of orders for 24-hour urine tests for our African American patients. Without 24-hour urine tests, African American patients are at a higher risk for stone recurrence than White patients. Separately, kidney stone analyses can provide information about the chemical composition of a patient’s stone. As with 24-hour urine tests, this information can help guide health care providers in the right direction when deciding on the proper dietary and pharmacologic therapies for the patient.

Biases 

Because this is a retrospective observational study without adjustment for socioeconomic, insurance, or access-related variables, alternative explanations for observed differences must be considered. Several unmeasured factors may have contributed to differences in diagnostic workup among patients with nephrolithiasis, including social determinants of health, documentation practices, and physician-level influences such as implicit bias. Implicit bias has been shown to negatively affect the quality of care provided to racial and ethnic minority patients [7]. Although not directly measured in this study, implicit bias remains a potential contributor to the observed disparities; however, its specific role cannot be determined from these data. The Implicit Association Test is a highly trusted and valid resource to assess an individual’s level of implicit bias [15]. One review study found that higher Implicit Association Test scores among health care providers resulted in lower satisfaction with clinical interaction for Black patients [16]. These findings suggest that tools such as the Implicit Association Test may have potential utility in helping physicians recognize their unintentional racial biases and potentially reduce observed differences in patient care. 

In addition to implicit bias, the exclusion of patients with missing demographic variables is a potential source of selection bias that could lead to reduced generalizability. Because of the small number of patients excluded (2.7%), the bias impact is expected to be minimal. Missing values in the analytical dataset are concentrated in the counseling variables, and if a stone analysis was performed. Kentucky's patient population in this cohort includes a largely rural and medically underserved population, which may further contribute to disparities in access to recommended testing, including 24-hour urine studies. 

Rural health and social determinants of health

The Appalachian region of Kentucky makes up a significant portion of the state and a large portion of the state’s rural area. Of the 120 counties in Kentucky, 85 are designated rural by the US Department of Agriculture [17]. In Appalachian Kentucky, there is a shortage in the supply of primary care physicians (PCPs) who provide health care to the region. The supply of PCPs per 100,000 population in this region is less than 26 percent of the national average [18]. Additionally, there is a shortage of specialty physicians. The supply of specialists per 100,000 population in Appalachian Kentucky is 59 percent lower than the national average [18]. The shortage of access to health care in the region may explain the higher burden of disease and poor health outcomes.

Moreover, most of Kentucky has some of the poorest health outcomes in the country. The burden of obesity and diabetes are just a few examples of poor health outcomes in the state. Kentucky is ranked 6th in the United States for diabetes mortality [19] and 9th for the prevalence of obesity [20]. The burden of these diseases is also higher in rural Appalachian Kentucky as opposed to other regions of Kentucky.

Obesity and diabetes are known risk factors for kidney stones [21]. Kentucky is a largely rural state, and rural areas tend to have higher rates of food insecurity. People who experience food insecurity typically have less access to fresh and nutritionally rich foods and more access to highly processed, nutrient-poor foods and foods that have higher levels of sodium [21]. Consumption of highly processed foods leads to a higher risk for the development of obesity and diabetes [22], both of which are risk factors for kidney stone formation. Implementing a policy that aims to reduce food insecurity in rural areas in Kentucky may be a possible avenue to reduce the incidence of nephrolithiasis.

There are opportunities to reduce the high incidence of nephrolithiasis in the state of Kentucky altogether by implementing policies to address the shortage of primary care physicians and specialty physicians in rural Kentucky. Additionally, implementing policies that can aim to reduce food insecurity in rural areas in Kentucky may be a possible avenue to reduce the incidence of nephrolithiasis. 

Limitations

Due to retrospective and observational design, our ability to determine causality is limited, and the observed differences in nephrolithiasis care by race should be interpreted as associations rather than direct effects. Accordingly, these findings are limited to differences in care processes and do not allow for the assessment of downstream clinical outcomes, such as stone recurrence or stone-free status. Furthermore, data were obtained through electronic record review, and some elements of care, such as dietary counseling or patient education, may have been provided but not documented, potentially leading to underestimation of care delivery. Variability in documentation practices and across participating sites may also have contributed to observed differences. Race was recorded as documented in the electronic health record and may be subject to misclassification in cases where race may have been assigned or assumed by clinical staff. Finally, this exploratory analysis did not adjust for potential confounding factors, such as socioeconomic status, insurance coverage, geographic access to care, or health literacy, which may contribute to observed differences. Additionally, the relatively small number of patients in certain racial minority groups may limit statistical power and the precision of subgroup comparisons; future multi-center studies or meta-analyses may help provide more robust estimates in underrepresented populations. Despite these limitations, this study highlights important differences in nephrolithiasis care and identifies opportunities for quality improvement and future investigation.

## Conclusions

Our findings identify differences in guideline-concordant nephrolithiasis care across racial groups. These differences likely reflect a combination of structural barriers, access to care, documentation practices, and potential physician-level factors. However, due to the observational nature of this study, causality cannot be established. These findings highlight important targets for quality improvement and warrant further investigation into underlying drivers of observed disparities; additionally, our findings underscore the importance of adherence to AUA guidelines for the medical management of nephrolithiasis care to promote consistency in nephrolithiasis evaluation and management. For future study, it would be beneficial to further examine the role of social determinants of health, healthcare access, and geographic barriers in shaping care delivery. Additionally, incorporating physician-level variables and multi-institutional data may identify factors that contribute to observed disparities and guide targeted interventions.
